# Assessing the Health and Functionality of the Microcirculation Using Thermal Imaging

**DOI:** 10.1002/jbio.70270

**Published:** 2026-05-14

**Authors:** Douglas B. Kell, Etheresia Pretorius

**Affiliations:** ^1^ Department of Biochemistry, Cell and Systems Biology, Institute of Systems, Molecular and Integrative Biology University of Liverpool Liverpool UK; ^2^ Department of Physiological Sciences, Faculty of Science Stellenbosch University Matieland South Africa

## Abstract

The microcirculation, composed of vessels below 100 μm in diameter, sustains tissue perfusion and metabolic exchange. Its dysfunction is increasingly implicated in chronic, inflammatory, and thrombotic disorders such as diabetes, sepsis, cardiovascular disease, and Long COVID. Accurate, noninvasive assessment of microvascular health is therefore clinically significant. Infrared thermal imaging provides a rapid, contact‐free, and physiologically coherent means of visualizing temperature distributions that reflect underlying blood flow. Because thermal gradients directly correspond to perfusion heterogeneity, this approach offers an interpretable surrogate for assessing microvascular function. Here, we review the physical principles of thermal imaging, summarize its application to the peripheral circulation, and compare it with established modalities including nailfold capillaroscopy and laser‐based techniques. We also outline its utility across diverse pathologies associated with fibrinaloid microclot complexes and endothelial injury. Thermal imaging thus emerges as an inexpensive/scalable tool for evaluating microcirculatory dysfunction in both research and (where approved) in clinical settings.

## Graphical abstract

We review the techniques of thermal imaging for assessing the functional consequences of lowered blood flow in the microcirculation that accompanies many chronic, inflammatory diseases. A comparison is made with laser Doppler/speckle methods.

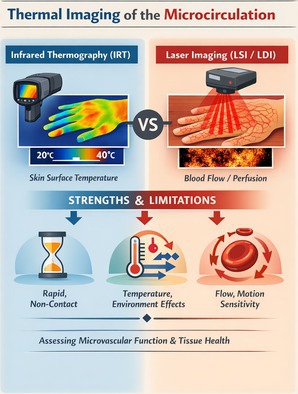

## 1 Introduction

The microcirculation is usually taken to refer to the flow of blood through microvessels with a diameter less than ~100 μm [1, 2, 3, 4, 5, 6, 7], and may be subject to significant remodeling [8]. As recently listed [9], a great many chronic, inflammatory diseases involve disorders of the microcirculation, which makes the noninvasive imaging of the microcirculation of considerable importance. We have recently reviewed the uses of nailfold (video)capillaroscopy [9] and both laser Doppler and laser speckle methods [7] for these purposes, particularly from the perspective of how the microcirculation may be disrupted by fibrinaloid microclot complexes [7, 9, 10, 11, 12, 13, 14, 15, 16, 17, 18, 19, 20].

**FIGURE 1:**
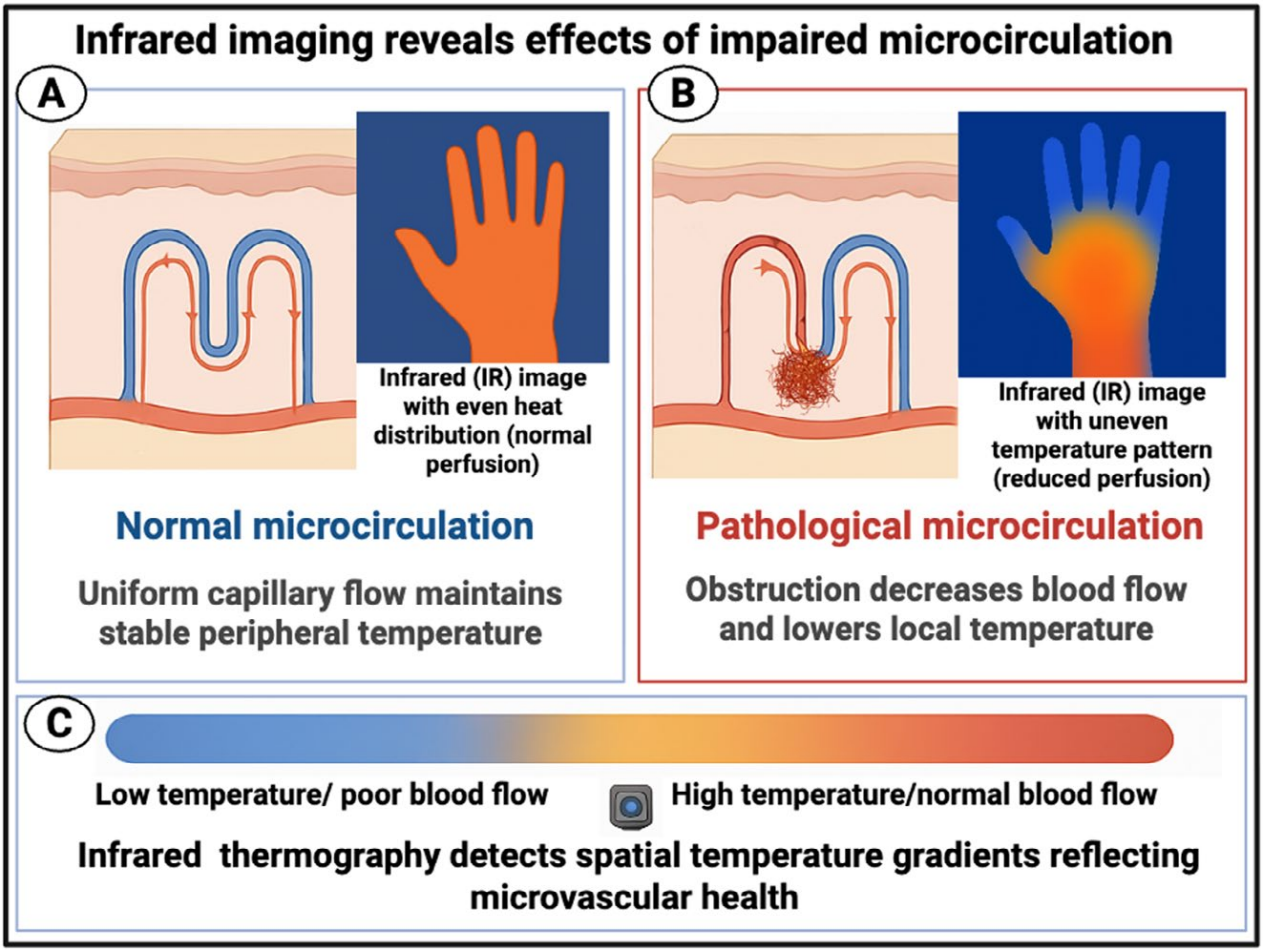
Infrared thermography provides a noninvasive method to visualize temperature gradients that correspond to underlying microvascular perfusion. (A) *Left panel:* In normal microcirculation, uniform capillary flow maintains stable peripheral temperature, resulting in an infrared (IR) image showing even heat distribution (orange coloration). (B) *Right panel:* In pathological microcirculation, obstructions or impaired capillary flow reduce perfusion and local heat emission, producing an uneven infrared temperature pattern with cooler regions (blue tones). (C) The temperature scale below illustrates the relationship between perfusion and heat distribution, where orange indicates normal blood flow and blue represents reduced perfusion or poor blood flow. Created in https://BioRender.com.

Since the temperature of blood at ca 37°C is normally greater than that of the ambient environment (and if not the latter can be lowered accordingly, or hands dipped into cold water), it is obvious that a decrease in peripheral blood flow might then manifest as a locally lowered temperature, and that measuring this, for instance via infrared radiation, could then serve to assess the microcirculation. Similarly, infection or inflammation might raise the temperature, whether locally or generally. Most pertinently, any lowering of blood flow increases thermal gradients, so the spatial assessment of temperature provides a clear indication of impaired blood flow (see Figure 1). Consequently, another class of noninvasive imaging method for assessing the microcirculation is therefore represented by infrared‐based imaging [21, 22, 23]. The purpose of the present article is thus to review this and to assess the extent to which it has been or might usefully be applied to diseases of the microcirculation and the effects of any treatments thereon. A preprint has been lodged [24].

## 2 Basis of Infrared Thermal Imaging

**FIGURE 2:**
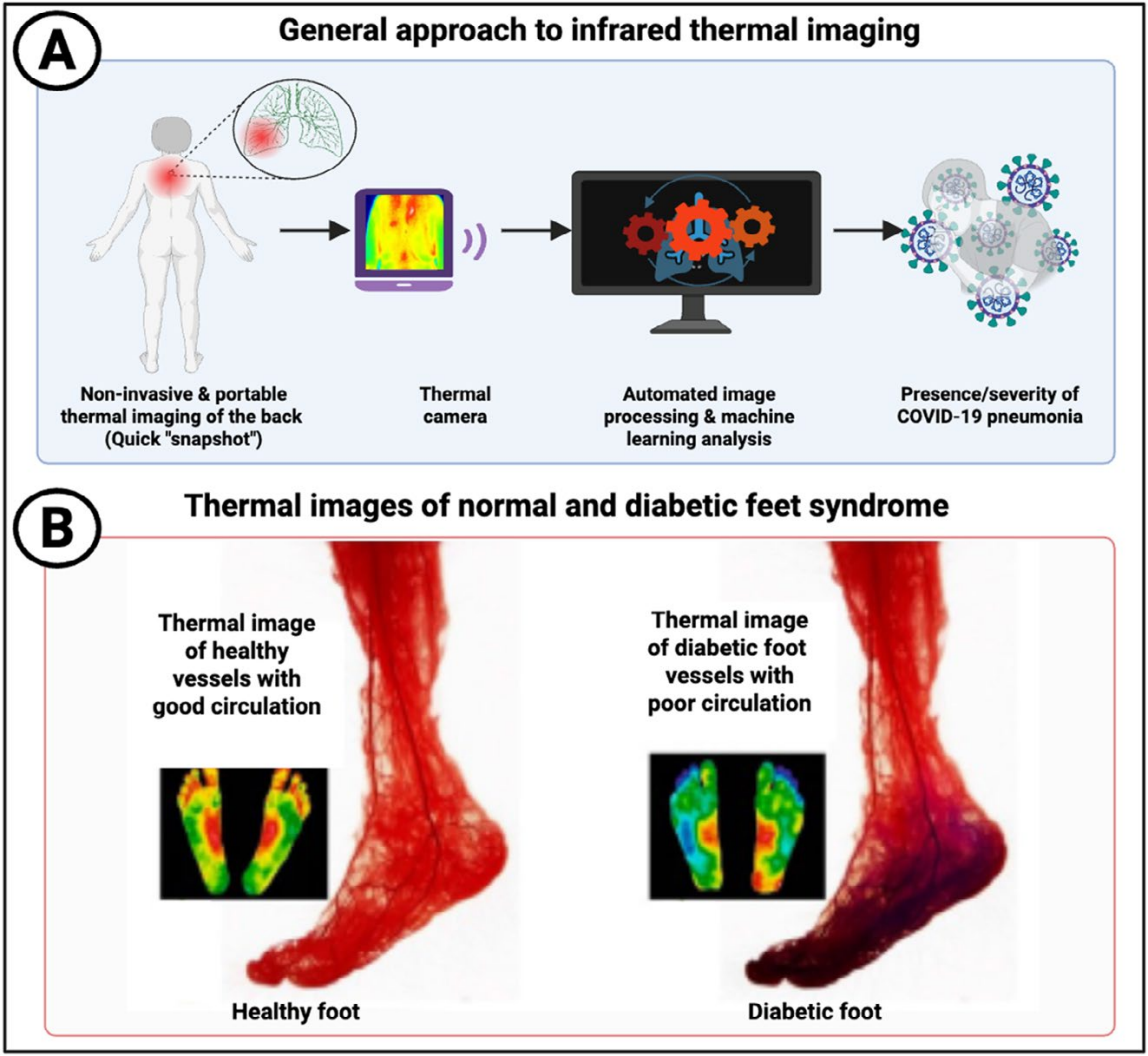
(A) General approach to thermal imaging, redrawn from a CC‐BY 4.0 publication [27] as applied to COVID‐19. Requirements are unhindered access to the target body's surface and a suitable thermal camera. The raw data are then processed and displayed using suitable algorithms, followed by an interpretational or predictive step (in this case the presence and/or severity of acute COVID‐19). (B) Color‐coded imaging data (red warm, blue cold) redrawn from a different CC‐BY 4.0 publication [31] illustrating the impaired circulation in diabetic feet. Created in https://BioRender.com.

Although other methods of thermography exist [25], we shall here concentrate on infrared imaging. All matter with a temperature above absolute zero emits radiation, mostly in the “thermal infrared band” (wavelength range 2–14 μm). If a material (surface) has a perfect emissivity (of 1) this is black‐body radiation. In practice, emissivities can vary, though that of human skin is fairly high [26], and this needs to be taken into account [27]. Note that it is not necessary to illuminate the target (this has its own bioeffects [28, 29]) as it emits the radiation itself. In the temperature range of interest, the intensity is proportional to the temperature while the peak is around 10 μm, shifting very slightly to shorter wavelengths as temperature increases. The general approach (taken from an example with COVID‐19 [30]) is illustrated in Figure 2, along with an example from normal vs diabetic feet.

While we are not seeking to recommend any particular system, thermal imaging cameras are widely available for £200–400, including as attachments for smartphones [32, 33, 34, 35, 36, 37, 38, 39, 40], and their resolution is typically better than 0.1°C in the range of present interest. Consequently we consider that these kinds of techniques might become more or less easily available.

## 3 Infrared Imaging of the Peripheral Circulation

We begin by mentioning some general reviews of thermal imaging for assessment of (patho)physiology in mammalian systems [27, 31, 36, 41, 42, 43, 44, 45, 46, 47, 48, 49, 50, 51, 52, 53, 54, 55, 56, 57, 58, 59, 60, 61, 62, 63, 64, 65, 66, 67]. Some focus explicitly on the microcirculation [66, 68, 69, 70, 71, 72, 73, 74] or on peripheral vascular systems [42, 53, 56, 59, 65, 69, 74, 75, 76, 77, 78, 79, 80, 81, 82, 83, 84, 85, 86, 87, 88, 89, 90, 91, 92]. From the numbers and scope of these reviews alone, it is clear that these methods have considerable potential, and so we now look (Table 1) at the variety of diseases of the microcirculation to which they have been applied. As before [9], we list in a separate column those diseases for which plasma fibrinaloid microclot complexes have been measured experimentally to be significantly above those of nominally healthy controls. In most cases the diagnosis is based on spatial differences reflecting (lack of) blood flow rather than absolute temperatures, while in some cases thermal flows are assessed temporally by imposing a cold challenge and looking for recovery.

**TABLE 1:** A series of diseases of the microcirculation along with details of whether they have been assessed via thermal imaging.

Disease or syndrome	Selected references using thermal imaging to assess the disease	Comments	References showing fibrinaloid microclot complexes (where tested)
**Acute COVID‐19**	[30, 35, 36, 93, 94, 95, 96, 97]	Thermal imaging was widely used in airports to detect fever rather than microcirculation issues [98, 99]. However, spatial imaging to detect deranged microcirculation was also used [30, 94, 95, 100]	[101, 102, 103, 104, 105, 106]
Acute respiratory distress syndrome	[107, 108]	Mostly thermodilution assays	
Age‐related macular degeneration (AMD)	[109, 110, 111]	Result can be a balance between inflammatory Temp increase and microcirculation‐based decrease	
**Alzheimer's dementia** (including mild cognitive impairment)	[74, 78, 112]	Also relates to functional capacity	[113, 114, 115, 116, 117]
Amyotrophic lateral sclerosis (ALS)	[118]	Large effect in the one example studied	
Antiphospholipid syndrome	[119]	Surprising lack of studies given its clear relation to microcirculation dysfunction	
Asthma	[120]		
Atopic dermatitis	[121, 122]	Surprisingly little recent literature	
Behçet's disease	[123]	A thermographic technique involving placing feet in a water bath at 42°C	
Cancers	[41, 124, 125, 126, 127, 128, 129]	Due to the significance of vascularization to tumor growth there is a massive literature. The list at left is purposely highly selective. However, there is necessarily a tendency for assessments to be localized	
Cardiovascular disease	[130, 131, 132, 133, 134, 135, 136]	Thermal imaging largely absent [137, 138]	
**Chronic fatigue syndrome**		Nothing as yet; huge opportunity!	[11, 139]
Chronic kidney disease	[140]	Closely related to cardiovascular complications	
Chronic obstructive pulmonary disease	[141, 142, 143]	Relatively little literature, but impaired microcirculation evident	
Chronic venous insufficiency	[65, 144, 145]	Can manifest, e.g., as leg ulcers	
Connective tissue disorders	[54, 71, 146, 147, 148]		
Deep vein thrombosis	[149, 150, 151, 152, 153, 154, 155, 156]	Very strong signals of thermal horspots	
Dengue fever	[98]		
Dermatology	[157]		
Diabetes mellitus, type 1	[158, 159, 160, 161]		
**Diabetes mellitus, type 2**	[111, 162, 163, 164, 165]		[106, 113, 166, 167, 168]
Diabetic foot and foot ulcers	[31, 34, 169, 170, 171, 172, 173, 174, 175, 176, 177]	Caused by derangment of the microcirculation; thermography is frequently used in diagnosis and prognosis	
Diabetic retinopathy	[111, 178, 179, 180]	Measurement of ocular surface temperature	
**Disseminated intravascular coagulation**		Astonishingly, given the links at right, seemingly nothing using thermal imaging—another massive opportunity in the ICU	[181] and see [182]; microparticles predicted DIC with an odds ratio exceeding 50 [181]
**Endothelial (dys)function generally**	[183, 184, 185, 186, 187, 188, 189, 190, 191, 192, 193]	Commonly done (as “vascular reactivity”) by studying thermal flow after a change in temperature or occlusion with a cuff	[194]
Erectile dysfunction	[68, 195, 196, 197]	Inadequate blood flow clearly closely involved	
Fibromyalgia	[148, 198, 199, 200, 201]	Quite variable results, and not yet seen as reliable	
Hypertension	[133, 193, 202, 203, 204, 205]	Most easily explained by increased resistance of microcirculation building up blood pressure [206]	
Influenza		No thermal imaging papers, though microcirculation clearly affected [207]	
Ischemic heart disease	[208]		
Leg ulcers	[209, 210, 211]	Related to chronic venous insufficiency. Textural analysis of images also useful	
**Long COVID**	[212, 213]	Surprisingly few given the ease of measurement and the well‐established derangement of the microcirculation	[12, 14, 16, 194, 214, 215, 216, 217, 218, 219, 220, 221]
Lupus (systemic lupus erythematosus, SLE)	[54, 71, 119, 222, 223]		
Lymphoedema	[224, 225, 226, 227, 228, 229]		
Malaria		While the microcirculation is strongly affected [230, 231] no thermal imaging papers seem to have been published	
Metabolic dysfunction‐associated steatotic liver disease (MASLD)	[232, 233]	More based on surface temperature measurements than the established microcirculation [234] effects	
Metabolic syndrome	[235, 236, 237, 238, 239]	Based on temperature differences between separate locations	
**Migraine**	[240, 241, 242, 243, 244, 245, 246, 247, 248]	Clear evidence of impaired perfusion in migraineurs, consistent with the microclot complexes observed	[249]
Multiple sclerosis	[250, 251]	Mostly studied in experimental autoimmune encephalomyelitis in mice. Note that thermoregulation is dysfunctional in MS [252, 253, 254]	
Obstructive sleep apnea	[255, 256, 257]	Observable signals but literature sparse	
Osteoarthritis	[54, 148, 258, 259, 260, 261]	Mostly detects inflammation. Standardization especially important incl equilibration to ambient temperature	
Pain in general	[262, 263, 264, 265, 266, 267]		
**Parkinson's disease**	[268, 269, 270, 271]	Clear evidence for impaired micrcirculation, consistent with fibrinaloid microclot complexes	[113, 272, 273, 274]
Peripheral artery disease	[275, 276, 277, 278, 279]	Best assessed spatially	
Podiatry	[280]		
Pre‐eclampsia		Surprisingly, given its etiology (e.g., [281, 282, 283, 284]), there seem to be no measurements using thermal imaging	
Psoriasis	[285, 286, 287]	Clear effects of inadequate perfusion	
Raynaud's phenomenon	[259, 288, 289, 290, 291, 292, 293, 294]	Thermography is seen as an effective method of assessment	
**Rheumatoid arthritis**	[54, 148, 259, 295]	Focus tends to be more on inflammation	[296, 297, 298]
Sarcoidosis	[222, 299]	Clear relation with poor perfusion but negligible recent literature	
**Sepsis and septic shock**	[90, 300, 301, 302, 303, 304]	Classic disease of the microcirculation [4, 305, 306, 307], thermography can be useful, but not always predictive of mortality. Also related to skin mottling. Capillary fill time and core‐to‐skin temperature gradient most predictive	[181] and see [182] (high predictive power for mortality)
Sickle cell disease	[186, 308, 309]	Includes leg ulcers as caused by hypoperfusion that Is a consequence of sickling	
Sjögren's syndrome	[148, 310]		
**Stroke (ischemic)**	[311, 312, 313, 314, 315, 316, 317]	Reasonably widely applied; stroke causes desymmetrization of temperature homogeneity seen in healthy controls	Amyloid observed in both microclot complexes [318] and macroclots [319, 320]
Systemic sclerosis (scleroderma)	[146, 288, 289, 292, 321, 322, 323, 324, 325, 326, 327, 328, 329]	Widely and effectively used here	
Transient ischemic attack (TIA)	[316, 330]	Usefully predictive	
Traumatic brain injury and other traumas	[40, 58, 331, 332, 333, 334, 335, 336, 337, 338, 339, 340, 341, 342, 343]	Very clear signals, including in assessment of burns and other wounds	
Vasculitis	[344, 345, 346]	Includes arteritis	

*Note:* Diseases where fibrinaloid microclot complexes have been reported are given in bold face.

## 4 Discussion

Our interest here relates to diseases that are associated with a decrease in or inhibition of the microcirculation, especially when this is caused by fibrinaloid microclot complexes. Specifically, we here add thermal imaging to the use of nailfold (video)capillaroscopy [9] and laser Doppler/speckle imaging methods [7]. Thermal methods have three particular attractions:

- they provide a straightforward, principled, and mechanistic understanding of the functional consequences of lowered blood flow,
- they are relatively inexpensive to implement: common cameras with software and interfaces to laptops or smartphones are available for £200–400, and
- unlike other methods inflammation can be observed separately via raised temperatures, although we recognize that this can be a confounder relative to the spatially lowered temperature caused by dysregulation of the microcirculation, adding a certain interpretational complexity.

Because we have covered these laser Doppler/speckle methods in considerable detail elsewhere [7], we just briefly rehearse the relative strengths and weaknesses of the two strategies (Table 2).

**TABLE 2:** A comparison of laser Doppler/speckle methods with thermal imaging.

Feature	Laser methods (LSCI/LDI)	Thermal imaging (IRT)
Measured variable	Microvascular perfusion/erythrocyte motion	Skin surface temperature
Relation to blood flow	Relatively direct	Indirect
Units	Relative perfusion units	°C
Acquisition speed	LSCI rapid; LDI slower scanning	Rapid
Field of view	Excellent full‐field perfusion maps	Excellent full‐field thermal maps
Depth	Superficial microcirculation	Skin surface only
Contact	Noncontact	Noncontact
Illumination	Requires laser illumination	Passive; no illumination needed
Motion artifact	Important limitation	Less affected by erythrocyte‐motion artifact, but still sensitive to movement and setup
Environmental dependence	Moderate	High
Physiological specificity	Higher for perfusion impairment	Lower; temperature reflects multiple influences
Use in challenge tests	Very good	Very good
Best applications	Perfusion mapping, vascular reactivity, heterogeneity screening	Thermal pattern mapping, rewarming studies, tissue viability, inflammation screening
Principal advantage	More direct functional assessment of perfusion	Simple, passive, rapid, intuitive
Principal limitation	Sensitive to motion; often semi‐quantitative	Indirect surrogate of perfusion

Compared with infrared thermography, laser speckle and laser Doppler methods provide a more direct functional readout of microvascular perfusion, since they depend on optical signatures of moving erythrocytes rather than on downstream thermal consequences at the skin surface. By contrast, thermography is entirely passive, very rapid, inexpensive and clinically intuitive, but it is intrinsically indirect: skin temperature reflects blood flow only in combination with heat transfer, thermoregulation, tissue properties and environmental conditions. Thus, laser methods are generally preferable when the aim is to quantify dynamic changes in perfusion or vascular reactivity, whereas thermal imaging is especially attractive as a complementary method for screening, regional mapping, and monitoring tissue viability, inflammation, ischemia, or rewarming responses. In particular, it is also very considerably cheaper (a few hundred pounds/dollars/Euros) vs. tens of thousands.

Unfortunately in the UK these methods seem not yet to have been approved for general clinical use, but in some cases have been approved as an ‘adjunctive measure’.

## 5 Conclusions

Infrared thermography provides a physiologically coherent and technically accessible method for assessing the functionality of the microcirculation. It complements existing optical and flow‐based imaging modalities by directly visualizing the thermal consequences of perfusion heterogeneity; thereby linking vascular function to measurable physiological outcomes. Its affordability, portability, and capacity for repeated, noncontact assessment position it as a promising tool for both clinical evaluation and longitudinal disease monitoring. When used alongside established modalities, such as nailfold capillaroscopy, laser‐based imaging, and biochemical profiling of fibrinaloid microclot complexes, thermal imaging may enable a more comprehensive appraisal of endothelial health and vascular dysregulation. Future work should focus on standardizing acquisition parameters, defining normative temperature baselines, and integrating thermal data with quantitative biomarkers of coagulation and inflammation. Such advances are likely to consolidate infrared thermography as an indispensable component of multimodal strategies aimed at elucidating, diagnosing, and ultimately mitigating microcirculatory dysfunction.

## Author Contributions

Conceptualization: Douglas B. Kell and Etheresia Pretorius. Formal analysis: Douglas B. Kell and Etheresia Pretorius. Resources: Douglas B. Kell and Etheresia Pretorius. Writing – original draft preparation: Douglas B. Kell and Etheresia Pretorius. Writing – review and editing: Douglas B. Kell and Etheresia Pretorius. Visualization: Douglas B. Kell and Etheresia Pretorius. Funding acquisition: Douglas B. Kell and Etheresia Pretorius.

## Funding

D.B.K. thanks the Balvi Foundation (grant 18) for funding. E.P. thanks the PolyBio Research Foundation and Kanro Foundation for funding. The content and findings reported and illustrated are the sole deduction, view, and responsibility of the researchers and do not reflect the official position and sentiments of the funders. The funders had no role in study design, data collection and analysis, decision to publish, or preparation of the manuscript.

## Conflicts of Interest

E.P. is a named inventor on a patent disclosing the use of fluorescence microscopy in Long COVID.

## Data Availability

Data sharing not applicable to this article as no datasets were generated or analysed during the current study.
